# Predictors and moderators of problem‐focused social skills training versus resource activation for children with disruptive behavior problems

**DOI:** 10.1002/jcv2.70170

**Published:** 2026-08-25

**Authors:** Leonie Hofmann, Christina Dose, Manfred Döpfner, Anja Görtz‐Dorten

**Affiliations:** ^1^ Faculty of Medicine and University Hospital Cologne Center for Child and Adolescent Cognitive Behavior Therapy (CEKIP) University of Cologne Cologne Germany; ^2^ Faculty of Medicine and University Hospital Cologne, Department of Child and Adolescent Psychiatry, Psychosomatics and Psychotherapy University of Cologne Cologne Germany

**Keywords:** child‐centered intervention, differential treatment effects, disruptive behavior disorder, moderation effects, personalized treatment, prediction analysis, randomized controlled trial

## Abstract

**Background:**

Not all children benefit equally from evidence‐based treatments for disruptive behavior disorders (DBD). In a previous randomized controlled trial (RCT), computer‐facilitated social skills training (ScouT) demonstrated superior efficacy over solution‐focused, resource‐oriented treatment (STARK) in reducing parent‐rated peer‐related aggression in children with DBD. This study explores predictors and moderators of these treatments to determine which subgroups respond differently.

**Methods:**

The sample included *N* = 100 children aged 6–12 years with DBD and peer‐related aggression. Using an exploratory approach, we selected potential predictors and moderators (child and sociodemographic characteristics, symptom severity, comorbid symptoms, parental distress, and treatment adherence) based on their associations with post‐treatment parent‐rated peer‐related aggression. We subsequently conducted hierarchical multiple regression analyses and single moderation analyses to identify predictors and moderators of the treatment effects.

**Results:**

Treatment adherence and most pre‐treatment child and family characteristics were not significant predictors or moderators. In moderation analyses, ScouT showed stronger effects than STARK on parent‐rated peer‐related aggression in children with low levels of parent‐rated internalizing symptoms and low levels of clinician‐rated DBD symptoms at pre‐treatment. No significant group differences were detected in children with high levels of these variables. In hierarchical multiple regression analyses, younger age, higher functional impairment, and higher internalizing symptoms at pre‐treatment predicted poorer outcomes, nonspecific to treatment type. Results differed across secondary analyses using teacher‐ and child‐rated outcomes.

**Conclusion:**

Moderation analyses support the robustness of RCT findings: ScouT's superior efficacy in reducing parent‐rated peer‐related aggression seems to hold across various characteristics. However, children with high levels of internalizing symptoms and high DBD symptom severity may need more targeted or longer treatment. The small effect sizes of the moderation effects limit the relevance of the findings for clinical practice. Age, functional impairment, and internalizing symptoms predict outcome irrespective of the treatment modality. These findings should inform future hypothesis‐testing studies.

## Key Points

**What's Known?**

- Research has shown that problem‐focused social skills training (ScouT) is superior to resource‐activating intervention (STARK) in reducing parent‐rated peer‐related aggression in children with disruptive behavior disorders (DBD).

**What's New?**

- In our study, treatment adherence and most pre‐treatment child and family characteristics were not significant predictors or moderators of peer‐related aggression.
- Children with low DBD and low internalizing symptoms benefited more from ScouT, whereas both interventions were similarly effective for children with higher levels.
- Younger age, higher functional impairment, and higher internalizing symptoms predicted poorer outcomes in both treatment modalities.

**What's Relevant?**

- ScouT's superior efficacy seems to hold across various characteristics.
- DBD symptoms, internalizing symptoms, functional impairment, and age may influence treatment and might be considered in clinical planning.

## INTRODUCTION

Although there is a range of well‐established or likely efficacious treatments for children with disruptive behavior disorders (DBD), including oppositional defiant disorder (ODD) and conduct disorder (CD; Kaminski et al., 2024), not all children benefit equally (Weisz et al., 2017). Identifying moderators of treatment effects may help determine which subgroups benefit more or less, thereby enabling the development of tailored interventions and optimizing treatment outcomes (Kraemer et al., 2002; McMahon et al., 2021).

Overall, findings on moderators of DBD treatment effects are heterogeneous (Selph et al., 2025). In meta‐analyses and reviews of different treatments for aggressive behaviors in children, only pre‐treatment symptom severity has been identified fairly consistently as a moderator, with stronger treatment effects reported in children with higher initial symptom severity (Hendriks et al., 2018; McMahon et al., 2021), potentially reflecting greater room for improvement as well as higher parental motivation (Leijten et al., 2018). For other variables, the findings are still tentative. For example, these treatments seem to be unaffected by child gender (Hendriks et al., 2018; Smeets et al., 2015) or diagnosis/diagnostic status and comorbidity (McMahon et al., 2021; Ollendick et al., 2008; Smeets et al., 2015). In contrast, the presence of paternal involvement and more severe maternal depressive symptoms, the latter likely reflecting greater baseline severity and therefore a greater capacity for change, may be associated with larger effects (McMahon et al., 2021). Although lower socioeconomic status (SES) might be expected to negatively affect treatment outcomes due to increased contextual stressors, the few existing findings are mixed, with studies indicating stronger effects in higher‐SES families, others in lower‐SES families, and some suggesting interactions with pre‐treatment aggression (Hendriks et al., 2018). Results are also highly inconsistent regarding age effects (Hendriks et al., 2018; Smeets et al., 2015).

Many previous studies focused on parent management training (PMT) and included nonclinical populations. Far less is known about the moderation effects in child‐centered interventions specifically for children with DBD (Hendriks et al., 2018; McMahon et al., 2021). Although PMT is the most established intervention for DBD (Gatti et al., 2019; Kaminski et al., 2024), child‐focused therapy may be particularly beneficial for improving children's social skills (Goertz‐Dorten, Benesch, et al., 2019; Webster‐Stratton et al., 2004). Given that moderation effects appear to vary across intervention types (McMahon et al., 2021), findings on PMT or combined treatments may not generalize to child‐centered approaches. Effects of child‐focused interventions may depend more strongly on child characteristics. For example, low cognitive abilities may hinder children's engagement with and responsiveness to the intervention, thereby limiting treatment effects (Dedousis‐Wallace et al., 2022; Mathiassen et al., 2012).

A meta‐analysis examining child‐centered cognitive behavioral therapy (CBT) found that the effects on anger experiences were stronger in mixed‐gender populations than in exclusively male populations, whereas no moderation effects of pre‐treatment symptoms or age were detected (Sukhodolsky et al., 2004). In contrast, another meta‐analysis of children and youth with antisocial behavior found that older participants benefited more from child‐centered CBT than younger participants did, with no effect of ethnicity (McCart et al., 2006). Similarly, Beelmann and Lösel (2021) found meta‐analytic evidence that the effects of social skills training were moderated by age and gender. However, the effects diminished in a multiple regression model including these moderators. Finally, a meta‐analysis examining the effectiveness of social skills training for juvenile delinquents found no moderating effects of age, gender, or ethnicity on training outcomes (van der Stouwe et al., 2021). None of these child‐centered studies investigated the impact of SES, comorbidity, or cognitive abilities.

Moderation analyses in studies comparing two active treatments are especially informative, as they may help identify optimal intervention settings for different subgroups (e.g., clinic‐based vs. community setting; Shelleby & Kolko, 2015), guide decisions about the suitable delivery format for specific populations (e.g., group‐based vs. internet‐delivered; Engelbrektsson et al., 2023), or elucidate which stakeholders should participate (e.g., PMT vs. parent‐ and child‐directed; Dedousis‐Wallace et al., 2022).

The current study therefore aimed to examine potential moderators in the comparison of two active, child‐centered interventions for children with DBD: the Computer‐Assisted Social Skills Training for Children with Aggressive Behavior (ScouT; German: Soziales computerunterstütztes Training für Kinder mit aggressivem Verhalten; Görtz‐Dorten & Döpfner, 2016), a problem‐focused intervention, and the Supportive Resource Activation Treatment (STARK; German: Supportive Therapie zur Aktivierung von Ressourcen bei Kindern und Jugendlichen; Perri et al., in press), which targets children's self‐esteem. A previous randomized controlled trial (RCT) demonstrated significant pre‐post improvements in both groups, with superiority of ScouT in improving parent‐rated peer‐related aggression in 100 children with DBD and peer‐related aggressive behaviors (6–12 years).

Given the inconsistent findings on moderation effects in DBD treatment and because, to the best of our knowledge, this is the first study to examine moderators in the comparison between computer‐facilitated, problem‐focused social skills training and a resource activation treatment, we followed the recommendation of Kraemer et al. (2006) to use exploratory moderation analyses. These may serve as a basis for future hypothesis‐testing studies. Because interaction effects are typically small and difficult to detect (von Hippel & Schuetze, 2025), limiting their clinical relevance, we also examined treatment‐nonspecific predictors (predictors independent of treatment modality). Specifically, we employed an inductive approach to data analysis and considered a broad set of potential predictors and moderators, including child characteristics, sociodemographic characteristics, symptom severity, comorbid symptoms, parental psychological distress, and child treatment adherence.

## MATERIAL AND METHODS

### Study design

The data for this study originate from a previous RCT comparing STARK and ScouT (Goertz‐Dorten et al., 2022). After an initial screening (T1a), eligible participants entered an 8‐week waiting period, which was relevant only for within‐subject analyses (see Goertz‐Dorten, Groth, et al., 2019). At the end of the waiting period, eligibility was reassessed (T1b), and participants still meeting the inclusion criteria were randomly allocated to ScouT (*n* = 50) or STARK (*n* = 50) via computerized block randomization. Both interventions comprised 16 individual 50‐min sessions for the children, supplemented by two psychoeducational sessions for the parents. Following the intervention, a post‐assessment (T2) was conducted. The children and parents were blinded to the specific research hypotheses, whereas the clinicians were not. For further details, see Goertz‐Dorten et al. (2022).

### Participants

The inclusion criteria were (1) an ICD‐10 diagnosis of ODD, CD (F91), mixed disorder of conduct and emotions (F92), or hyperkinetic CD (F90.1), established via clinician‐administered parent interviews using the Diagnostic Checklist for Disruptive Behavior Disorders (DCL‐DBD; Döpfner et al., 2008), (2) children’s aggressive behavior toward peers, assessed using the DCL‐DBD, (3) elevated symptoms of DBD at T1b, indicated by a stanine score ≥ 7 on the parent‐rated German Symptom Checklist for Disruptive Behavior Disorders (SCL‐DBD; Döpfner et al., 2008), (4) age 6–12 years, and (5) IQ ≥ 80 on the Culture Fair Intelligence Test (Weiß, 2006; Weiß & Osterland, 2012).

The exclusion criteria were the presence of a severe comorbid disorder (e.g., autism) requiring priority treatment, planned changes in any pre‐existing psychotropic medication, participation in another child psychotherapy, self‐reported severe mental disorders in the parents (clinical assessment) that were considered to potentially interfere with study participation (e.g., questionnaire completion, regular treatment attendance), and insufficient German‐language proficiency of the parents.

Between July 2014 and February 2016, 141 children referred to the outpatient unit of the Center for Child and Adolescent Cognitive Behavior Therapy (CEKIP) at the University Hospital Cologne, Germany, were screened for study participation. Twenty participants were excluded during the initial screening phase (T1a), and 21 participants no longer met the inclusion criteria after the 8‐week waiting period (T1b), resulting in a final sample of 100 children. Written informed consent was obtained from all participants and their parents.

### Interventions

ScouT is a child‐centered, therapist‐led, computer‐assisted, problem‐focused social skills training program for children with aggressive behavior, which was applied during face‐to‐face treatment sessions (Görtz‐Dorten & Döpfner, 2016). It uses a problem‐solving approach and integrates cognitive‐behavioral techniques, including video modeling, role‐playing, and homework assignments, and features video vignettes. These vignettes present five peer conflict scenarios: disappointment, verbal aggression, physical aggression, nonacceptance of responsibility, and devaluation. After watching the videos, the children work through problem‐solving exercises to examine thoughts and emotions, evaluate consequences, and generate alternative responses. Therapists guide these tasks in each session, support self‐reflection, and help transfer the skills to real‐life situations.

STARK is a child‐centered, transdiagnostic, solution‐oriented intervention for children that focuses on resource activation (Perri et al., in press). It includes four modules: (1) psychoeducation, therapeutic relationship, motivation, and goal setting; (2) identifying personal resources and enhancing self‐esteem, self‐acceptance, and self‐confidence; (3) applying these resources to achieve daily goals and improve well‐being, including relaxation and mindfulness; and (4) consolidating these resources through therapist and family support, including resource‐oriented exercises.

The degree of therapist guidance was comparable between ScouT and STARK.

### Measures

Most measures were assessed immediately prior to treatment (pre‐treatment; T1b) as well as following the 16‐session treatment (post‐treatment; T2). For organizational reasons, the DCL‐DBD was applied at the beginning of the 8‐week waiting period (pre‐treatment; T1a). Treatment adherence was assessed after each of the 16 sessions between T1b and T2. For continuous measures, scale scores were computed by averaging the corresponding item scores. The term parent refers to the primary caregiver.

#### Outcome: Peer‐related aggression

Parents, teachers, and children rated peer‐related aggression using the 25‐item German‐language Questionnaire for Aggressive Behavior of Children (FAVK; Görtz‐Dorten & Döpfner, 2010). The FAVK comprises four subscales for peer‐related aggression (disturbance of social‐cognitive information processing, social skills, impulse control, and social interaction), which are combined into the FAVK Total Peer score. The items are rated on a scale from 0 (“not at all”) to 3 (“very much”). Elevated parent‐rated aggression was associated with the following mean scale scores: 0.68 for children < 9 years and 0.64 for children ≥ 9 years. The four‐factor structure has been validated using confirmatory factor analyses (Benesch et al., 2013). In our sample, the FAVK Total Peer score showed good internal consistency for parent‐, teacher‐, and self‐ratings (*α* = .89 for each rating).

#### Potential predictors and moderators

##### Child and sociodemographic characteristics

We recorded the child's biological sex, age, IQ, and medication status (yes/no), the educational level of the parent with the highest education, and the child's living arrangement (living with two vs. one or no primary caregivers [e.g., single parent, residential placement]).

##### Symptom severity and functional impairment

Parents rated DBD symptoms based on ICD‐10 and DSM‐IV criteria using the SCL‐DBD (Döpfner et al., 2008). Its items are rated on a scale from 0 (“not at all”) to 3 (“very much”). The 25 subscale items for ODD and CD are combined into the SCL‐DBD Total score. Previous research has confirmed the factorial validity of the SCL‐DBD (Görtz‐Dorten et al., 2014; Ise et al., 2014). In our sample, the SCL‐DBD Total score showed acceptable internal consistency (*α* = .72). Clinicians rated DBD symptoms using the DCL‐DBD on an identical 0–3 scale (Döpfner et al., 2008). To ensure comparability with the parent ratings, the ODD and CD subscales with 15 items were combined to form the DCL‐DBD Total score. The DCL‐DBD has demonstrated factorial validity (Döpfner et al., 2008). In our sample, the DCL‐DBD Total score had a low Cronbach's *α* of .63.

Parents rated callous‐unemotional (CU) traits using the 24‐item Inventory of Callous‐Unemotional Traits (ICU; Frick, 2003). The items are rated on a scale ranging from 0 (“not at all true”) to 3 (“definitely true”). Analyses of the factorial structure revealed the best model fit for a general CU factor with three independent factors (Benesch et al., 2014). In our sample, the ICU Total score demonstrated good internal consistency (*α* = .82). Additionally, parents rated their children's functional impairment using the modified 31‐item German version of the Weiss Functional Impairment Rating Scale‐Parent Report (WFIRS‐P‐M; Kernder et al., 2019). The items were rated on a scale from 0 (“never or not at all”) to 3 (“very often or very much”). The factorial structure of the subscales and the general impairment factor have been empirically validated (Kernder et al., 2019). The Cronbach's *α* of the WFIRS Total score was good in our sample (*α* = .88).

##### Comorbid symptoms

Parents rated child internalizing symptoms using the Internalizing Problems scale from the German version of the Child Behavior Checklist for Ages 6–18 (CBCL 6–18; Döpfner et al., 2014). Items were rated on a scale ranging from 0 (“not true”) to 2 (“very true or often true”). The factorial validity of the scale has been confirmed (Döpfner et al., 2014). In the present sample, the CBCL Internalizing Problems score demonstrated acceptable internal consistency (*α* = .77). Additionally, parents rated ADHD symptoms based on ICD‐10 and DSM‐IV criteria using the 20‐item Symptom Checklist for Attention‐Deficit/Hyperactivity Disorder (SCL‐ADHD; Döpfner et al., 2008) on a scale from 0 (“not at all”) to 3 (“very much”). The SCL‐ADHD has demonstrated reliability and factorial validity (Brühl et al., 2000; Erhart et al., 2008). In our sample, the Cronbach's *α* of the SCL‐ADHD Total score was excellent (*α* = .94).

##### Parental psychological distress

Parents rated their psychological distress using the 42‐item self‐report Depression Anxiety Stress Scales (DASS; Essau, 1995). The items are rated on a scale from 1 (“never”) to 4 (“very often”). The factorial structure has been validated in international studies (Brown et al., 1997; Lovibond & Lovibond, 1995). In our sample, the DASS Total score had an excellent Cronbach's *α* (*α* = .94).

##### Child treatment adherence

Clinicians rated child adherence on a newly developed Adherence Checklist (ADCH). With four items, this checklist assessed cooperation during the sessions and adherence to therapeutic homework assignments on a scale ranging from 0 (“not at all”) to 3 (“very much”). Scores were averaged across all 16 sessions. The internal consistency of this score was good in the current sample (*α* = .83).

### Statistical analyses

The analyses were conducted using SPSS version 30. For pre‐ and post‐treatment data, we calculated scale scores for participants with less than 10% missing items. This applied to the parent‐ and child‐rated FAVK and to the baseline scores of the scales used as predictors and moderators. However, for the post‐treatment teacher‐rated FAVK in the STARK group, missing data were imputed using the expectation‐maximization method with available STARK pre‐ and post‐treatment data as predictors. No imputation was performed for variables that were assessed only at pre‐treatment (e.g., sociodemographic information) or for the process‐related variable treatment adherence.

First, we examined the intercorrelations among all potential predictors and moderators, including child characteristics (age, IQ, medication), sociodemographic characteristics (highest parental educational attainment, living arrangement, number of children in the current household), symptom severity and functional impairment (parent‐rated SCL‐DBD Total, ICU Total and WFIRS Total, clinician‐rated DCL‐DBD Total), comorbid symptoms (parent‐rated CBCL Internalizing and SCL‐ADHD Total), parental psychological distress (parent‐rated DASS Total), and child treatment adherence (clinician‐rated ADCH). Collinearity was considered problematic at |*r*| > 0.70 (Dormann et al., 2013).

For all subsequent analyses, the primary outcome measure was parent‐rated peer‐related aggression at post‐assessment (FAVK Total Peer), consistent with the primary outcome of the RCT by Goertz‐Dorten et al. (2022). The self‐report version of the FAVK is only suitable for children aged 9 years and older, whereas our sample included a substantial proportion of younger children; moreover, parent reports may reduce social desirability bias and provide a broader cross‐situational assessment of behavior compared to teacher reports. Nevertheless, secondary analyses using child‐ and teacher‐rated FAVK Total Peer scores as outcomes are provided in the Supporting Information. All analyses controlled for the FAVK Total Peer pre‐treatment scores to adjust for potential baseline differences.

We followed an inductive approach, selecting potential predictors and moderators based on their association with the outcome. For this purpose, we ran separate regression models for each treatment group and for each variable of interest, predicting post‐treatment peer‐related aggression from each potential predictor and moderator, controlling for pre‐treatment parent‐rated peer‐related aggression. Variables with *p* < .05 in at least one group were retained for further hierarchical regression and moderation analyses. Due to our inductive approach, we did not correct for multiple testing (Kraemer et al., 2002).

To identify treatment‐nonspecific predictors of the treatment outcome, we conducted hierarchical multiple regression analyses. The variables were entered following theoretical proximity to the outcome and according to prior evidence on prognostic factors in DBD (e.g., Hendriks et al., 2018; McMahon et al., 2021): Steps 1–2: control variables (treatment group and pre‐treatment outcome score); Step 3: symptom severity and functional impairment; Step 4: comorbid symptoms; Step 5: child characteristics; Step 6: sociodemographic characteristics; Step 7: parental distress; Step 8: treatment adherence. We report standardized regression coefficients (*β*) to allow for a comparison of the relative strengths of the predictors and the adjusted *R* ^2^ to account for the number of predictors and the sample size, providing a more conservative estimate (Cohen et al., 2013).

We subsequently performed moderation analyses using the PROCESS macro 4.3 for SPSS (Hayes & Little, 2022). Moderation means that the effect of an independent variable *X* on a dependent variable *Y* is influenced and predicted by a moderator variable *W* (Hayes & Little, 2022). To identify moderators of treatment effects, the significance of the interaction between the independent variable (here: treatment group) and the respective moderator is of interest (Hayes & Little, 2022). Each potential moderator was examined in a separate analysis to clearly identify the individual interaction effect between the treatment group (STARK = 0, ScouT = 1) and the respective moderator on reducing peer‐related aggression.

PROCESS uses ordinary least squares regression to estimate model parameters. We report unstandardized regression coefficients and bias‐corrected bootstrap confidence intervals with 5000 resamples as well as the proportion of variance in *Y* uniquely attributed to the moderation of *X*'s effect by *W* (Δ*R* ^2^) and *f* ^2^ $\left(f^{2}=\frac{\Delta R^{2}}{1-R_{\text{included}}^{2}}\right)$ as effect size indicators of the interaction (Cohen, 1988; Hayes & Little, 2022). For significant continuous moderators, we used the Johnson–Neyman technique to probe values at which the conditional effect of *X* on *Y* transitions between significant and nonsignificant (Bauer & Curran, 2005).

## RESULTS

### Sample characteristics, pre‐treatment group comparisons, and intercorrelations

Table 1 presents sample and pre‐treatment data of potential predictors and moderators. There were no significant group differences, with the exception that ScouT participants presented significantly higher parent‐rated FAVK Total Peer scores of peer‐related aggression. However, this score was used as a control variable in all analyses. The small number of nonmale participants precluded an analysis of sex as a potential predictor and moderator.

**TABLE 1:** Pre‐treatment demographic and clinical characteristics of ScouT and STARK participants.

Variable		ScouT		STARK		*χ* ^2^	df	*p*
	*N*	*n*	%	*n*	%			
Child and sociodemographic characteristics								
Sex	100					0.15	1	.695
Male		46	92	47	94			
Female		4	8	3	6			
Diagnoses	100					0.84	2	.658
Oppositional defiant disorder (ICD‐10: F91.3)		33	66	34	68			
Conduct disorder (ICD‐10: F91.1 and F91.2)		9	18	6	12			
Hyperkinetic conduct disorder (ICD‐10: F90.1)		8	16	10	20			
Medication^a^	96					0.59	1	.442
Yes		8	16	11	22			
Living arrangement^b^	98					1.54	1	.214
One or no primary		6	12	11	22			
Caregiver								

	***N***	***M***	**SD**	***M***	**SD**	***t***	**df**	***p***
Child age in years	100	9.50	1.84	9.96	1.89	1.22	97.95	.226
IQ	100	100	14	104	14	1.48	97.96	.142
Highest parental educational attainment^c^	93	2.54	1.05	2.40	1.21	−0.60	87.25	.550
Number of children in current household	96	1.96	1.04	2.15	0.86	0.98	92.00	.332
Symptom severity and functional impairment								
Parent‐rated FAVK total peer	100	1.62	0.43	1.32	0.44	−3.39	97.94	.001
Parent‐rated SCL‐DBD total	100	0.86	0.24	0.79	0.20	−1.71	94.38	.090
Parent‐rated ICU total	100	1.22	0.45	1.15	0.32	−0.83	88.74	.409
Clinician‐rated DCL‐DBD total	100	0.93	0.25	0.96	0.24	0.63	97.81	.532
Parent‐rated WFIRS total	100	0.99	0.40	0.97	0.41	−0.22	97.96	.825
Comorbid symptoms								
Parent‐rated CBCL internalizing^d^	100	0.33	0.21	0.32	0.24	−0.16	96.64	.876
Parent‐rated SCL‐ADHD total	100	1.34	0.65	1.43	0.64	0.76	97.96	.447
Parental psychological distress								
Parent‐rated DASS total	100	1.51	0.39	1.45	0.30	−0.78	92.48	.437

Abbreviations: CBCL = Child Behavior Checklist, DASS = Depression Anxiety Stress Scales, DCL‐DBD = Diagnostic Checklist for Disruptive Behavior Disorders, df = degrees of freedom, FAVK = Questionnaire for Aggressive Behavior of Children, ICU = Inventory of Callous‐Unemotional Traits, *M* = mean, *N*/*n* = sample size, *p* = significance value, SCL‐ADHD = Symptom Checklist for Attention‐Deficit/Hyperactivity Disorder, SCL‐DBD = Symptom Checklist for Disruptive Behavior Disorders, ScouT = Computer‐Assisted Social Skills Training for Children with Aggressive Behavior, SD = standard deviation, STARK = resource activation treatment, *t* = empirical *t*‐value, WFIRS‐P‐M = Weiss Functional Impairment Rating Scale–Parent Report, modified version, *χ* ^2^ = empirical *χ* ^2^ value.

^a^ 0 = no, 1 = yes (all received stimulant medication; one child additionally received antipsychotics).

^b^ 0 = living with one or no primary caregiver (e.g., single parent, residential placement), 1 = living with two primary caregivers.

^c^ 0 = no qualifications, 1 = lower‐track secondary school, 2 = medium‐track secondary school, 3 = higher‐track secondary school, 4 = university degree.

^d^ Gender‐ and age‐normed *T*‐scores: ScouT *M* = 60.20, SD = 8.42; STARK *M* = 59.30, SD = 10.02.

Children in ScouT attended a mean of 15.66 treatment sessions (SD = 1.29; range = 8–16) and children in STARK 15.96 sessions (SD = 0.28; range = 14–16). Clinician‐rated adherence was high in both groups but significantly higher in ScouT than STARK (ScouT: *M* = 2.48, SD = 0.42, two missing values; STARK: *M* = 2.30, SD = 0.48, one missing value; *t* = −2.00, df = 93.45, *p* = .049).

Intercorrelations between potential predictors and moderators ranged from *r* = .00 to *r* = .66, and therefore did not indicate multicollinearity (Supporting Information S1: Table S1).

### Exploring potential predictors of post‐treatment peer‐related aggression in STARK and ScouT

All regression models were conducted separately for each predictor within each treatment group and controlled for pre‐treatment outcome scores. None of the sociodemographic variables, medication, parent‐rated DBD symptoms and CU traits, as well as child treatment adherence, significantly predicted post‐treatment parent‐rated peer‐related aggression in either STARK or ScouT (Table 2). Therefore, these variables were excluded from the subsequent regression and moderation analyses. In both groups, higher pre‐treatment parent‐rated functional impairment and comorbid symptoms predicted higher post‐treatment peer‐related aggression. Only in STARK did higher age and IQ predict lower peer‐related aggression, whereas only in ScouT did higher clinician‐rated DBD symptoms and parent‐rated parental psychological distress predict higher peer‐related aggression.

**TABLE 2:** Prediction of post‐treatment parent‐rated peer‐related aggression (FAVK Total Peer) separately for STARK and ScouT (*N* = 100).

Predictors	ScouT		STARK	
	*β*	*p*	*β*	*p*
Child characteristics				
Age	−.04	.749	−.34	.008
IQ	.01	.955	−.29	.022
Medication^a^ ^,^ ^b^	.18	.161	.12	.375
Sociodemographic characteristics				
Highest parental educational attainment^c^ ^,^ ^d^	−.02	.869	−.07	.637
Living arrangement^e^ ^,^ ^f^	−.21	.090	.00	.997
Number of children in current household^g^	−.15	.236	.12	.414
Symptom severity				
Parent‐rated SCL‐DBD total	.04	.798	.30	.068
Parent‐rated ICU total	−.13	.408	.17	.221
Parent‐rated WFIRS total	.44	<.001	.28	.044
Clinician‐rated DCL‐DBD total	.30	.027	−.04	.748
Comorbid symptoms				
Parent‐rated CBCL internalizing	.45	<.001	.30	.038
Parent‐rated SCL‐ADHD total	.31	.022	.37	.003
Parental symptoms				
Parent‐rated DASS total	.28	.028	.15	.272
Child treatment adherence				
Clinician‐rated ADCH^h^	−.13	.338	−.14	.287

*Note*: Most potential predictors and moderators were assessed at pre‐treatment; only adherence reflects the mean across all 16 sessions. All analyses were conducted separately for each predictor and FAVK Total Peer scores at pre‐treatment served as a covariate in each model.

Abbreviations: *β* = standardized regression coefficient, ADCH = Adherence Checklist, CBCL = Child Behavior Checklist, DASS = Depression Anxiety Stress Scales, DCL‐DBD = Diagnostic Checklist for Disruptive Behavior Disorders, FAVK = Questionnaire for Aggressive Behavior of Children, ICU = Inventory of Callous‐Unemotional Traits, *p* = significance level, SCL‐ADHD = Symptom Checklist for Attention‐Deficit/Hyperactivity Disorder, SCL‐DBD = Symptom Checklist for Disruptive Behavior Disorders, ScouT = Computer‐Assisted Social Skills Training for Children with Aggressive Behavior, STARK = resource activation treatment, WFIRS‐P‐M = Weiss Functional Impairment Rating Scale–Parent Report, modified version.

^a^ 0 = no, 1 = yes.

^b^ ScouT *n* = 48, STARK *n* = 48.

^c^ 0 = no qualifications, 1 = lower‐track secondary school, 2 = medium‐track secondary school, 3 = higher‐track secondary school, 4 = university degree.

^d^ ScouT *n* = 48, STARK *n* = 45.

^e^ 0 = living with one or no primary caregiver (e.g., single parent, residential placement), 1 = living with two primary caregivers.

^f^ ScouT *n* = 48, STARK *n* = 50.

^g^ ScouT *n* = 49, STARK *n* = 47.

^h^ ScouT *n* = 48, STARK *n* = 49.

While controlling for pre‐treatment outcome scores, living with two primary caregivers predicted lower post‐treatment teacher‐rated peer‐related aggression in ScouT, whereas higher parental educational attainment predicted higher post‐treatment child‐rated peer‐related aggression in STARK. No further significant predictors were identified (Supporting Information S1: Tables S2 and S3).

### Identifying treatment‐nonspecific predictors of post‐treatment peer‐related aggression

In the first step of the hierarchical multiple regression, the treatment group did not significantly predict post‐treatment parent‐rated peer‐related aggression (Table 3). Adding pre‐treatment peer‐related aggression in the second step significantly improved the model, explaining an additional 22% of the variance (adjusted *R* ^2^), and both variables significantly predicted peer‐related aggression. Including symptom severity and functional impairment in the third step increased the explained variance by 11% (adjusted *R* ^2^), with functional impairment emerging as an additional significant predictor. Adding parent‐rated comorbid symptoms in the fourth step added 7% explained variance (adjusted *R* ^2^), with internalizing and ADHD symptoms emerging as additional significant predictors. However, the association between functional impairment and peer‐related aggression dropped to nonsignificance in this model. In step five, age and IQ explained an additional 4% of the variance (adjusted *R* ^2^), and group, pre‐treatment peer‐related aggression, functional impairment, internalizing symptoms, and age were significant predictors. As no sociodemographic variables were relevant, parental psychological distress was entered in step 6 but did not significantly contribute to the explained variance. This model explained 44% of the variance (adjusted *R* ^2^) in parent‐rated peer‐related aggression, 22% more than group and pre‐treatment peer‐related aggression (control variables). Being in the ScouT group and being older predicted lower outcome scores, whereas higher parent‐rated peer‐related problems, functional impairment, and internalizing symptoms predicted higher scores. Clinician‐rated DBD symptoms, parent‐rated ADHD symptoms, IQ, and parent‐rated parental distress were not significant predictors.

**TABLE 3:** Hierarchical multiple regression to predict post‐treatment parent‐rated peer‐related aggression (FAVK total peer; *N* = 100).

Model		*b*	SE	95% CI		*β*	*p*
				LL	UL		
1	Intercept	1.05	0.08	0.90	1.20		<.001
	Group^a^	−0.14	0.11	−0.36	0.07	−.13	.188
	adj. *R* ^2^ = .008, adj. Δ*R* ^2^ = .008, Δ*F* (1, 98) = 1.76, *p* = .188						
2	Intercept	0.26	0.16	−0.06	0.58		.114
	Group^a^	−0.32	0.10	−0.52	−0.12	−.30	.002
	Parent‐rated FAVK total peer	0.60	0.11	0.38	0.82	.50	<.001
	adj. *R* ^2^ = .227, adj. Δ*R* ^2^ = .219, Δ*F* (1, 97) = 28.88, *p* < .001						
3	Intercept	−0.10	0.21	−0.51	0.32		.644
	Group^a^	−0.26	0.10	−0.45	−0.07	−.24	.008
	Parent‐rated FAVK total peer	0.39	0.12	0.15	0.62	.32	.001
	Clinician‐rated DCL‐DBD total	0.20	0.20	−0.20	0.59	.09	.322
	Parent‐rated WFIRS total	0.46	0.12	0.22	0.71	.34	<.001
	adj. *R* ^2^ = .331, adj. Δ*R* ^2^ = .112, Δ*F* (2, 95) = 8.47, *p* < .001						
4	Intercept	−0.11	0.20	−0.50	0.29		.593
	Group^a^	−0.22	0.09	−0.40	−0.04	−.21	.017
	Parent‐rated FAVK total peer	0.31	0.12	0.08	0.53	.26	.009
	Clinician‐rated DCL‐DBD total	0.11	0.19	−0.27	0.49	.05	.562
	Parent‐rated WFIRS total	0.22	0.14	−0.04	0.49	.17	.100
	Parent‐rated SCL‐ADHD total	0.16	0.08	0.01	0.32	.19	.041
	Parent‐rated CBCL internalizing	0.60	0.23	0.16	1.05	.25	.009
	adj. *R* ^2^ = .401, adj. Δ*R* ^2^ = .070, Δ*F* (2, 93) = 6.55, *p* = .002						
5	Intercept	1.17	0.51	0.15	2.19		.025
	Group^a^	−0.28	0.09	−0.46	−0.10	−.26	.003
	Parent‐rated FAVK total peer	0.29	0.11	0.06	0.51	.24	.013
	Clinician‐rated DCL‐DBD total	0.10	0.19	−0.27	0.47	.05	.593
	Parent‐rated WFIRS total	0.32	0.13	0.06	0.59	.24	.018
	Parent‐rated SCL‐ADHD total	0.06	0.09	−0.11	0.23	.07	.492
	Parent‐rated CBCL internalizing	0.68	0.22	0.25	1.12	.28	.003
	Age	−0.07	0.02	−0.12	−0.02	−.23	.006
	IQ	−0.01	0.00	−0.01	0.00	−.13	.117
	adj. *R* ^2^ = .440, adj. Δ*R* ^2^ = .039, Δ*F* (2, 91) = 4.30, *p* = .016						
6	Intercept	0.98	0.54	−0.08	2.05		.070
	Group^a^	−0.29	0.09	−0.48	−0.11	−.27	.002
	Parent‐rated FAVK total peer	0.29	0.11	0.07	0.51	.24	.012
	Clinician‐rated DCL‐DBD total	0.12	0.19	−0.25	0.49	.06	.511
	Parent‐rated WFIRS total	0.31	0.14	0.04	0.58	.23	.025
	Parent‐rated SCL‐ADHD total	0.03	0.09	−0.14	0.21	.04	.693
	Parent‐rated CBCL internalizing	0.66	0.22	0.22	1.09	.27	.004
	Age	−0.07	0.02	−0.11	−0.02	−.23	.008
	IQ	−0.01	0.00	−0.01	0.00	−.14	.102
	Parent‐rated DASS total	0.15	0.13	−0.10	0.41	.10	.242
	adj. *R* ^2^ = .443, adj. Δ*R* ^2^ = .003, Δ*F* (1, 90) = 1.39, *p* = .242						

*Note*: All predictors were assessed at pre‐treatment. Pre‐treatment parent‐rated FAVK Peer Total served as a covariate in all models.

Abbreviations: *β* = standardized regression coefficient, adj. *R* ^2^ = adjusted proportion of explained variance, adj. Δ*R* ^2^ = adjusted incremental variance, *b* = unstandardized regression coefficient, CBCL = Child Behavior Checklist, DASS = Depression Anxiety Stress Scales, DCL‐DBD = Diagnostic Checklist for Disruptive Behavior Disorders, FAVK = Questionnaire for Aggressive Behavior of Children, Group = STARK (0) versus ScouT (1), LL = lower limit, *p* = significance level, SCL‐ADHD = Symptom Checklist for Attention‐Deficit/Hyperactivity Disorder, SE = standard error, UL = upper limit, WFIRS = Weiss Functional Impairment Rating Scale–Parent Report, modified version.

^a^ 0 = STARK, 1 = ScouT.

In the final models, living arrangement was not a significant treatment‐nonspecific predictor of teacher‐rated peer‐related aggression, whereas parental educational attainment was a significant predictor of child‐rated peer‐related aggression, explaining an additional 9% of the variance (adjusted *R* ^2^; Supporting Information S1: Tables S4 and S5).

### Identifying moderators of the treatment effects on parent‐rated peer‐related aggression

**FIGURE 1:**
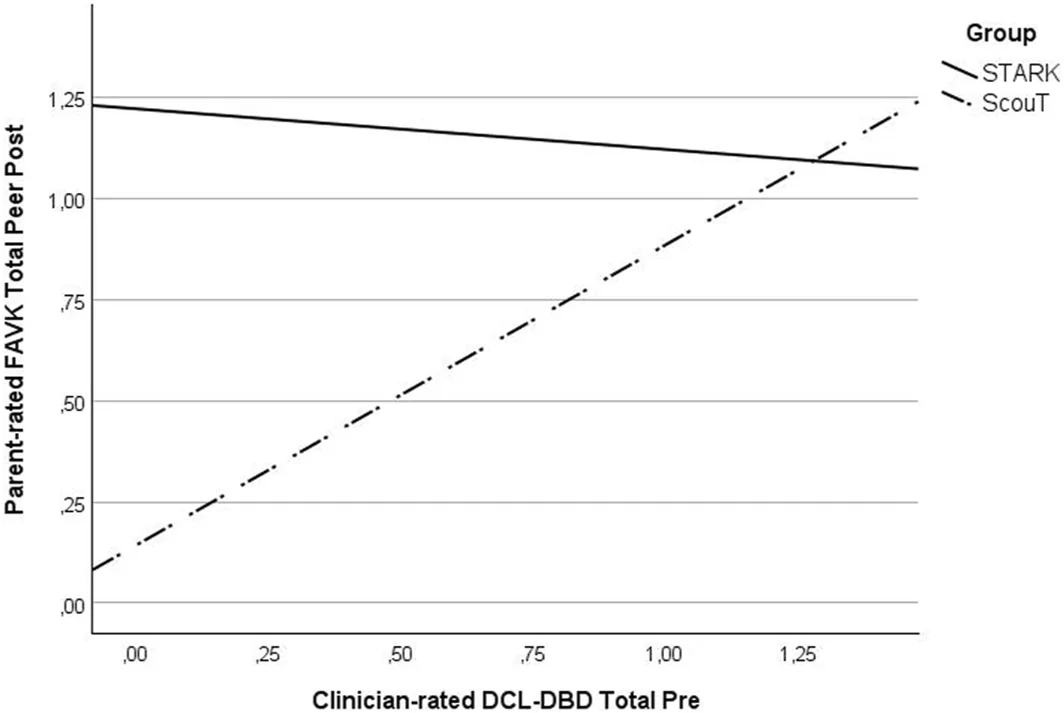
Pre‐treatment clinician‐rated DBD symptoms as a moderator of the effects of ScouT compared to STARK on post‐treatment parent‐rated peer‐related aggression (FAVK Total Peer; *N* = 100). The pre‐treatment parent‐rated FAVK Total Peer served as a covariate. DCL‐DBD = Diagnostic Checklist for Disruptive Behavior Disorders, FAVK = Questionnaire for Aggressive Behavior of Children, ScouT = Computer‐Assisted Social Skills Training for Children with Aggressive Behavior, STARK = Resource Activation Treatment.

**FIGURE 2:**
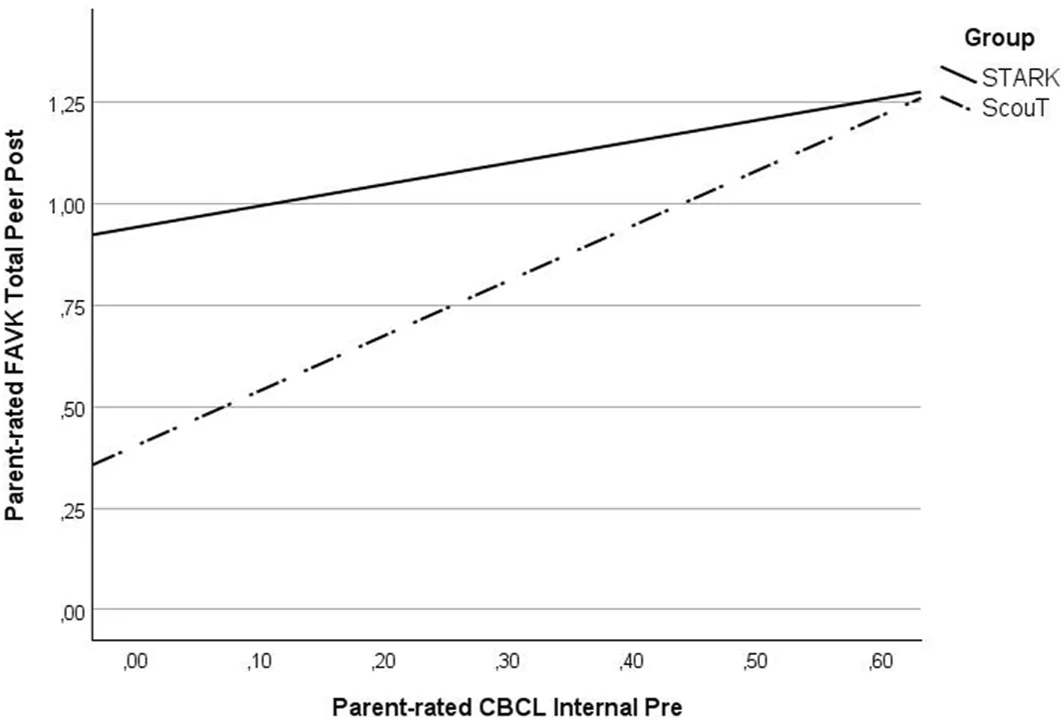
Pre‐treatment parent‐rated internalizing symptoms as a moderator of the effects of ScouT compared to STARK on post‐treatment parent‐rated peer‐related aggression (FAVK Total Peer; *N* = 100). The pre‐treatment parent‐rated FAVK Total Peer served as a covariate. CBCL = Child Behavior Checklist, FAVK = Questionnaire for Aggressive Behavior of Children, ScouT = Computer‐Assisted Social Skills Training for Children with Aggressive Behavior, STARK = Resource Activation Treatment.

Controlling peer‐related aggression at pre‐treatment, moderation analyses yielded a significant interaction between group and pre‐treatment clinician‐rated DBD symptoms and between group and pre‐treatment parent‐rated child internalizing symptoms. The interaction accounted for 4% (DBD symptoms as moderator) and 3% (internalizing symptoms as moderator) of the variance in parent‐rated peer‐related aggression. Probing the interaction per model showed that ScouT was more effective than STARK in reducing parent‐rated peer‐related aggression in children with low clinician‐rated DBD symptoms (DCL‐DBD Total < 1.03; 72% of the sample) and low parent‐rated internalizing symptoms (CBCL Internalizing < 0.41; 66% of the sample), whereas no group differences were observed in children with more severe symptoms (see Figures 1 and 2). No other variables moderated the treatment effects (Table 4). Full results for the other coefficients in the model are provided in the Supporting Information (Supporting Information S1: Table S6).

**TABLE 4:** Moderation of the effects of ScouT compared to STARK on post‐treatment parent‐rated peer‐related aggression (FAVK total peer; *N* = 100).

	*b*	SE	*t*	*p*	95% CI		*f* ^2^
					LL	UL	
Child characteristics							
Group × age	0.06	0.05	1.24	.218	−0.04	0.16	0.02
Group × IQ	0.01	0.01	1.67	.098	−0.00	0.03	0.03
Symptom severity							
Group × parent‐rated SCL‐DBD total	−0.08	0.45	−0.17	.864	−0.98	0.82	0.00
Group × clinician‐rated DCL‐DBD total	0.85	0.39	2.19*	.031	0.08	1.61	0.05
Group × WFIRS total	0.37	0.22	1.71	.091	−0.06	0.81	0.03
Comorbid symptoms							
Group × parent‐rated CBCL internalizing	0.83	0.40	2.10*	.039	0.04	1.61	0.05
Group × parent‐rated SCL‐ADHD total	0.03	0.14	0.20	.842	−0.26	0.31	0.00
Parental psychological distress							
Group × parent‐rated DASS total	0.22	0.28	0.79	.432	−0.34	0.79	0.01

*Note*: All potential moderators were assessed before treatment. Pre‐treatment parent‐rated FAVK Total Peer served as a covariate in all analyses.

Abbreviations: *b* = unstandardized coefficient for the interaction effect, CBCL = Child Behavior Checklist, CI = confidence interval, DASS = Depression Anxiety Stress Scales, DCL‐DBD = Diagnostic Checklist for Disruptive Behavior Disorders, *f* ^2^ = effect size, FAVK = Questionnaire for Aggressive Behavior of Children, Group = STARK (0) versus ScouT (1), LL = lower limit, *p* = significance value, Pre = pre‐treatment, SCL‐ADHD = Symptom Checklist for Attention‐Deficit/Hyperactivity Disorder, SCL‐DBD = Symptom Checklist for Disruptive Behavior Disorders, ScouT = Computer‐Assisted Social Skills Training for Children with Aggressive Behavior, SE = standard error, STARK = Resource Activation Treatment, *t* = empirical *t*‐value, UL = upper limit, WFIRS‐P‐M = Weiss Functional Impairment Rating Scale–Parent Report, modified version.

^*^ *p* < .05.

For the teacher‐rated outcome, a significant interaction between group and living arrangement accounted for 5% of the variance (Supporting Information S1: Table S7). While neither conditional effect was statistically significant, there was a tendency for STARK to be more effective than ScouT in reducing peer‐related aggression among children living with one or no primary caregiver (*b* = 0.59, *p* = .055), whereas a reversed effect was observed for children living with two primary caregivers (*b* = −0.20, *p* = .140; Supporting Information S1: Figure S1). However, parental educational attainment did not moderate the treatment effects on child‐rated peer‐related aggression (Supporting Information S1: Table S8).

## DISCUSSION

This exploratory study examined predictors and moderators of the problem‐focused, computer‐assisted social skills training versus the resource‐activating intervention STARK in children with DBD, adding nuance to the main RCT finding that ScouT was superior to STARK in reducing parent‐rated peer‐related aggression (Goertz‐Dorten et al., 2022).

Most variables were not significant predictors or moderators of the treatment effects on parent‐rated peer‐related aggression. Specifically, most sociodemographic variables, medication, parent‐rated CU traits, and DBD symptoms, as well as child treatment adherence, were not significant predictors in the separate regression analyses. Although lower SES has been linked to higher levels of externalizing behavior (Peverill et al., 2021; Piotrowska et al., 2015), the findings on SES or related variables and treatment outcomes remain inconsistent (Engelbrektsson et al., 2023; Hendriks et al., 2018; Shelleby & Kolko, 2015). Concerning CU traits, in a meta‐analysis, affected children presented with higher symptom severity before and after treatment than did those with DBD alone, but nevertheless showed similar reductions in DBD symptoms (Perlstein et al., 2023). Aligning with this last finding, in our study, treatment outcome, when controlling for pre‐treatment scores, did not appear to be influenced by CU traits.

Besides the treatment group and pre‐treatment outcome as covariates, age, functional impairment, and internalizing symptoms were identified as significant nonspecific predictors of post‐treatment peer‐related aggression in multiple hierarchical regression. Across both treatment groups, older children showed lower peer‐related aggression after treatment. Similarly, several studies on child‐centered interventions have reported larger effects in older participants (Beelmann & Lösel, 2021; McCart et al., 2006), which may reflect more advanced cognitive skills. Nonetheless, some studies reported no predictive value of age (Sukhodolsky et al., 2004; van der Stouwe et al., 2021; van Stam et al., 2014). Stronger parental involvement may enhance treatment effects in younger children, consistent with evidence on parent‐directed interventions with or without child involvement (e.g., Kaminski et al., 2024).

Additionally, in our study, children with greater functional impairment and more internalizing symptoms benefited less, regardless of the treatment modality. Functional impairment refers to the extent to which symptoms interfere with everyday functioning across domains such as family life, academic performance, and social relationships (Kernder et al., 2019; Stein & Jessop, 1990). Although functional impairment is related to symptom severity, the two are distinct constructs (Rapee et al., 2012), and greater impairment may independently limit treatment gains. In line with this, the “complexity hypothesis” argues that children with more severe and comorbid presentations respond worse, as most interventions are developed for less complex cases (Kazdin & Whitley, 2006). On the other hand, research on mixed interventions (e.g., multimodal or multicomponent) for DBD tends to indicate that internalizing symptoms may not substantially affect treatment outcomes (Aitken et al., 2018; Masi et al., 2013; Ollendick et al., 2008), and the impact of functional impairment seems to be heterogeneous (Kolko & Pardini, 2010; Masi et al., 2013; Shelleby & Kolko, 2015). However, child‐centered formats may be sensitive to these clinical features, suggesting that addressing internalizing problems and functional impairment alongside DBD could be beneficial.

Only a few moderators of the treatment effects were identified. In conclusion, ScouT's superior effect on parent‐rated peer‐related aggression holds across a range of child and family characteristics (age, IQ, functional impairment, ADHD symptoms, and parental distress). However, clinician‐rated DBD symptoms and parent‐rated child internalizing symptoms acted as moderators of the treatment effects on peer‐related aggression.

Children with low DBD symptoms seem to benefit more from ScouT than from STARK, whereas both interventions seem similarly less effective in children with high DBD symptom levels. These children may require more targeted or longer interventions or may benefit from stronger parental involvement, as these children may be less likely to actively engage in child‐centered interventions. Similarly, Dedousis‐Wallace et al. (2022) reported poorer outcomes of PMT and combined parent‐child interventions in children with higher pre‐treatment conduct problems. On the other hand, some studies suggest stronger PMT effects in children with more severe symptoms (e.g., Hendriks et al., 2018; McMahon et al., 2021), possibly due to greater scope for improvement and higher parental motivation (Leijten et al., 2018). The present findings should be considered in light of the limited parental involvement. Future studies should examine in which cases stronger parental involvement may be particularly beneficial. Moreover, Sukhodolsky et al. (2004) reported that symptom severity did not moderate child‐centered CBT effects but found the strongest gains for moderate symptom levels compared with low and high levels. Unlike many prevention samples with low baseline symptoms, which were considered in most previous studies, clinical samples such as ours show consistently elevated symptoms. This may impact the efficacy of child‐centered interventions. Interestingly, parent‐rated DBD symptoms did not predict or moderate the treatment effects. Furthermore, the interpretability of the clinician ratings is limited by the low reliability of the DCL‐DBD Total score.

Besides their predictive effects, internalizing symptoms also appear to moderate the effects of ScouT versus STARK. Children with low internalizing symptoms benefited more from ScouT than from STARK, whereas both interventions seem similarly less effective in children with high symptom levels. It is notable that STARK, with its focus on resources, self‐esteem, and mindfulness, might be considered closer to interventions targeting internalizing difficulties. However, this does not necessarily translate into superior effects on peer‐related aggression.

While ScouT appears more effective than STARK in reducing peer‐related aggression, this effect may not hold for children with high DBD or internalizing symptoms. However, the clinical implications of our analyses are limited, given the small sizes of the moderating effects (*f* ^2^ = 0.05; Δ*R* ^2^ = 3%–5%). On average, effect sizes in moderation analyses tend to be even smaller (Aguinis et al., 2005), making it difficult for a single moderator to yield clinically meaningful effects (Mullarkey & Schleider, 2021). Future studies should use larger samples and therefore may consider multiple moderators simultaneously, as patients in real‐world settings typically present with complex profiles and outcomes are likely influenced by several interacting factors. Our hierarchical multiple regression analyses suggest that nonspecific predictors may be informative in explaining treatment outcomes, with the final model explaining 22% of the variance beyond the covariates. This indicates that factors such as age, baseline functional impairment, and internalizing symptoms, while not specific to the type of intervention, can meaningfully account for treatment outcomes and may help improve the precision of outcome predictions when incorporated into treatment planning.

For teacher‐ and child‐rated outcomes, fewer significant effects were observed, and findings were not consistent with those for parent‐rated outcomes. However, child self‐reports were restricted to participants aged ≥ 9 years (*n* = 67), limiting comparability with the parent‐rated findings. Moreover, because most potential predictors and moderators were parent‐rated, the ability to detect associations with teacher‐ and child‐rated outcomes may have been reduced. Limited agreement across informants is frequently observed in child mental health research, potentially reflecting, at least in part, meaningful context‐specific differences (De Los Reyes et al., 2015). Indeed, correlations between parent‐, teacher‐, and child‐rated outcomes were low or even nonsignificant in the present sample (see Supporting Information S1: Table S1). Given the aforementioned limitations of the analyses including teacher‐ and child‐rated peer‐related aggression, future research should further examine predictors and moderators of teacher‐ and child‐rated outcomes.

The present study has several limitations. First, the high proportion of boys reflects the general male preponderance in DBD but limits generalizability to other sexes and precluded the analysis of sex as a predictor or moderator. Second, ethnicity was not assessed according to current standards and could not be examined. Third, the analyses were exploratory, which increases the risk of type I error. However, this approach is appropriate for generating new insights and informing future confirmatory research (Kraemer et al., 2006). Fourth, the small sample size limited the power to detect smaller interaction effects, meaning that nonsignificant results cannot be regarded as clearly generalizable (see McMahon et al., 2021). Nevertheless, the present study provides first insights into predictors and moderators of problem‐oriented versus resource‐activation treatment for DBD, which can inform future studies with larger samples.

## CONCLUSION

Most child, family, and adherence characteristics were not meaningful predictors or moderators of parent‐rated peer‐related aggression. Overall, ScouT appeared to be more effective than STARK across diverse clinical profiles. Moderation analyses suggest that ScouT is more effective than STARK in children with low levels of internalizing symptoms and low DBD symptom severity, whereas children with high symptom levels benefit less and may need more targeted or longer treatment. However, the effect sizes of the moderation effects were small, limiting the clinical relevance of the findings. Nonspecific predictors may offer explanatory power for understanding treatment outcomes. Younger age, higher functional impairment, and higher internalizing symptoms may be associated with poorer outcomes, regardless of treatment type. Results varied for teacher‐ and child‐rated outcomes. These findings can inform future research and support clinical decision‐making in the long term. The analyses should be replicated in larger, more diverse samples.

## AUTHOR CONTRIBUTIONS

**Leonie Hofmann**: Writing—original draft; methodology; writing—review and editing; formal analysis. **Christina Dose**: Methodology; supervision; writing—review and editing. **Manfred Döpfner**: Supervision; writing—review and editing; methodology; conceptualization. **Anja Görtz‐Dorten**: Investigation; supervision; project administration; conceptualization; writing—review and editing; methodology.

## CONFLICT OF INTEREST STATEMENT

Anja Görtz‐Dorten and Manfred Döpfner authored the treatment manuals and some of the rating scales used for evaluation, for which they receive royalties from publishing companies. Anja Görtz‐Dorten has received income as head, supervisor, and lecturer of the Center for Child and Adolescent Cognitive Behavior Therapy (CEKIP) and as a consultant for child behavior therapy at the National Association of Statutory Health Insurance Physicians (Kassenärztliche Bundesvereinigung). She has received royalties from publishing companies (Hogrefe, Huber) as an author of treatment manuals, books, and rating scales to assess mental disorders, and of psychological tests, computer programs, and smartphone applications. She has served as an advisor for digital health applications, for which she has received royalties from a company (eyelevel GmbH) and research support from the German Ministry of Education and Research. Manfred Döpfner has received consulting income and research support from Medice, eyelevel GmbH, brainjo, and medigital, and research support from the German Research Foundation, the German Ministry of Education and Research, the German Ministry of Health, and the Innovation Fund. He has received income as head, supervisor, and lecturer of the Center for Child and Adolescent Cognitive Behavior Therapy (CEKIP) and as a consultant for child behavior therapy at the National Association of Statutory Health Insurance Physicians (Kassenärztliche Bundesvereinigung). He has received royalties from treatment manuals, books, and psychological tests published by Beltz, Elsevier, Enke, Guilford, Hogrefe, Huber, Kohlhammer, Schattauer, Springer, and Wiley. Christina Dose has received royalties from a publishing company (Hogrefe) as an author of self‐help books for parents and teachers of children with ADHD. The remaining authors have declared that they have no competing or potential conflicts of interest.

## ETHICAL CONSIDERATIONS

The study received ethical approval (Ethics Committee of the University Hospital Cologne, Germany, July 3, 2014, reference number: 13‐060). All procedures followed the ethical standards of the 1964 Declaration of Helsinki and its later amendments. Written informed consent was obtained from all participants and their parents, and they consented to the use of their data for academic publications.

## Supporting information

Supporting Information S1

## Data Availability

The data that support the findings of this study are available from the corresponding author upon reasonable request.
