# Supplementary material for: Predictors and moderators of problem‐focused social skills training versus resource activation for children with disruptive behavior problems
Source: JCPP Adv. 2026 Aug 25:e70170. Online ahead of print. doi: 10.1002/jcv2.70170 (PMC13507639; doi:10.1002/jcv2.70170)
Supplement: Supplementary file 1 [file JCV2-9999-e70170-s001.docx]

**Predictors and Moderators of Problem-Focused Social Skills Training versus Resource Activation for Children with Disruptive Behavior Problems**

**Supporting Information**

**Table S1** Intercorrelations Among Outcomes, Potential Predictors, and Potential Moderators (*N* = 100)

**Table S2** Prediction of Post-Treatment Teacher-Rated Peer-Related Aggression (FAVK Total Peer) Separately for STARK and ScouT (*N* = 100)

**Table S3** Prediction of Post-Treatment Child-Rated Peer-Related Aggression (FAVK Total Peer) Separately for STARK and ScouT (*N* = 67)

**Table S4** Hierarchical Multiple Regression to Predict Post-Treatment Teacher-Rated Peer-Related Aggression (FAVK Total Peer; *N* = 98)

**Table S5** Hierarchical Multiple Regression to Predict Post-Treatment Child-Rated Peer-Related Aggression (FAVK Total Peer; *N* = 63)

**Table S6** Moderation of the Effects of ScouT Compared to STARK on Post-Treatment Parent-Rated Peer-Related Aggression (FAVK Total Peer; *N* = 100)

**Table S7** Moderation of the Effects of ScouT Compared to STARK on Post-Treatment Teacher-Rated Peer-Related Aggression (FAVK Total Peer; *N* = 98)

**Figure S1** Living Arrangement as a Moderator of the Effects of ScouT Compared to STARK on Teacher-Rated Peer-Related Aggression (FAVK Total Peer; *N* = 98)

**Table S8** Moderation of the Effects of ScouT Compared to STARK on Post-Treatment Child-Rated Peer-Related Aggression (FAVK Total Peer; *N* = 63)

Table S1 Intercorrelations Among Outcomes, Potential Predictors, and Potential Moderators (*N* = 100)

|  |  | 1. | 2. | 3. | 4. | 5. | 6. | 7. | 8. | 9. | 10. |  | 11. | 12. | 13. | 14. | 15. | 16. | 17. | 18. |
| --- | --- | --- | --- | --- | --- | --- | --- | --- | --- | --- | --- | --- | --- | --- | --- | --- | --- | --- | --- | --- |
| 1. | Sex | 1 |  |  |  |  |  |  |  |  |  |  |  |  |  |  |  |  |  |  |
| 2. | Age | -.11 | 1 |  |  |  |  |  |  |  |  |  |  |  |  |  |  |  |  |  |
| 3. | IQ | -.04 | -.15 | 1 |  |  |  |  |  |  |  |  |  |  |  |  |  |  |  |  |
| 4. | Medication^1,a^ | .14 | .06 | .12 | 1 |  |  |  |  |  |  |  |  |  |  |  |  |  |  |  |
| 5. | Highest parental educational attainment^2,b^ | -.03 | -.06 | .31* | .01 | 1 |  |  |  |  |  |  |  |  |  |  |  |  |  |  |
| 6. | Living arrangement^3,c^ | -.12 | -.04 | .09 | -.02 | .27* | 1 |  |  |  |  |  |  |  |  |  |  |  |  |  |
| 7. | Number of children in current household^d^ | .10 | .08 | .06 | -.07 | .04 | .24* | 1 |  |  |  |  |  |  |  |  |  |  |  |  |
| 8. | Parent-rated SCL-DBD Total | -.04 | -.08 | -.04 | .14 | .03 | -.04 | .11 | 1 |  |  |  |  |  |  |  |  |  |  |  |
| 9. | Parent-rated ICU Total | .14 | -.12 | .06 | .09 | .09 | .13 | .10 | .39* | 1 |  |  |  |  |  |  |  |  |  |  |
| 10. | Clinician-rated DCL-DBD Total | .10 | -.10 | .03 | .35* | -.17 | -.09 | .03 | .46* | .17 | 1 |  |  |  |  |  |  |  |  |  |
| 11. | Parent-rated WFIRS Total | .09 | .06 | .04 | .21* | .16 | -.10 | -.20* | .40* | .26* | .29* |  | 1 |  |  |  |  |  |  |  |
| 12. | Parent-rated CBCL Internalizing | .01 | .02 | .10 | .26* | .12 | .13 | -.10 | .23* | .25* | .19 |  | .47* | 1 |  |  |  |  |  |  |
| 13. | Parent-rated SCL-ADHD Total | .13 | -.23* | -.19 | .22* | -.09 | -.06 | -.16 | .34* | .31* | .33* |  | .50* | .33* | 1 |  |  |  |  |  |
| 14. | Parent-rated DASS Total | .08 | -.14 | .01 | .01 | .18 | .13 | -.18 | .15 | .03 | .03 |  | .27* | .23* | .34* | 1 |  |  |  |  |
| 15. | Clinician-rated ADCH^e^ | -.06 | .34* | -.12 | -.24* | -.01 | -.01 | -.07 | -.05 | -.05 | -.08 |  | -.13 | -.26* | -.18 | -.14 | 1 |  |  |  |
| 16. | Parent-rated FAVK Total Peer | .03 | -.16 | .12 | .04 | -.01 | .04 | .04 | .66* | .49* | .29* |  | .39* | .39* | .21* | .13 | -.05 | 1 |  |  |
| 17. | Teacher-rated FAVK Total Peer | -.06 | -.24* | .08 | -.16 | .05 | -.19 | .00 | .10 | -.06 | .05 |  | -.01 | .00 | -.07 | -.00 | -.05 | .26* | 1 |  |
| 18. | Children-rated FAVK Total Peer^f^ | -.06 | -.18 | -.21 | -.16 | .03 | -.20 | -.01 | .17 | -.08 | -.01 |  | .21 | .27* | .22 | .06 | .12 | .23 | .20 | 1 |

*Note:* ADCH = Adherence Checklist, CBCL = Child Behavior Checklist, DASS = Depression Anxiety Stress Scales, DCL-DBD = Diagnostic Checklist for Disruptive Behavior Disorders, FAVK = Questionnaire for Aggressive Behavior of Children, ICU = Inventory of Callous-Unemotional Traits, SCL-ADHD = Symptom Checklist for Attention-Deficit/Hyperactivity Disorder, SCL-DBD = Symptom Checklist for Disruptive Behavior Disorders, ScouT = Computer-Assisted Social Skills Training for Children with Aggressive Behavior, STARK = Resource Activation Treatment, WFIRS-P-M = Weiss Functional Impairment Rating Scale–Parent Report, modified version. *n* = sample size, *r* = correlation coefficient. * *p* < .05.

^1^ 0 = no, 1 = yes

^2^ 0 = no qualifications, 1 = lower-track secondary school, 2 = medium-track secondary school, 3 = higher-track secondary school, 4 = university degree.

^3^ 0 = living with one or no primary caregiver (e.g., single parent, residential placement), 1 = living with two primary caregivers.

^a^ ScouT *n* = 48, STARK *n* = 48. ^b^ ScouT *n* = 48, STARK *n* = 45. ^c^ ScouT *n* = 48, STARK *n* = 50. ^d^ ScouT *n* = 49, STARK *n* = 47. ^e^ ScouT *n* = 48, STARK *n* = 49. ^f^ ScouT *n* = 33, STARK *n* = 34.

Table S2 Prediction of Post-Treatment Teacher-Rated Peer-Related Aggression (FAVK Total Peer) Separately for STARK and ScouT (*N* = 98)

|  | ScouT | | STARK | |
| --- | --- | --- | --- | --- |
| Predictors | *β* | *p* | *β* | *p* |
| Child characteristics |  |  |  |  |
| Age | 0.21 | .125 | -0.18 | .218 |
| IQ | -0.17 | .223 | -0.26 | .072 |
| Medication^1, a^ | 0.09 | .523 | 0.22 | .147 |
| Sociodemographic characteristics |  |  |  |  |
| Highest parental educational attainment^2, b^ | -0.10 | .482 | 0.05 | .732 |
| Living arrangement^3, c^ | -0.31 | .022 | 0.00 | .991 |
| Number of children in current household^d^ | -0.26 | .057 | -0.03 | .855 |
| Symptom severity |  |  |  |  |
| Parent-rated SCL-DBD Total | 0.00 | .986 | -0.05 | .757 |
| Parent-rated ICU Total | 0.16 | .234 | 0.23 | .118 |
| Parent-rated WFIRS Total | 0.24 | .066 | -0.08 | .592 |
| Clinician-rated DCL-DBD Total | 0.06 | .669 | -0.07 | .625 |
| Comorbid symptoms |  |  |  |  |
| Parent-rated CBCL Internalizing | 0.26 | .051 | -0.05 | .727 |
| Parent-rated SCL-ADHD Total | 0.17 | .198 | 0.19 | .192 |
| Parental symptoms |  |  |  |  |
| Parent-rated DASS Total | 0.14 | .301 | -0.00 | .989 |
| Child Treatment Adherence |  |  |  |  |
| Clinician-rated ADCH^e^ | -0.15 | .275 | -0.19 | .204 |

*Note:* Most potential predictors and moderators were assessed at pre-treatment; only adherence reflects the mean across all 16 sessions. All analyses were conducted separately for each predictor and FAVK Total Peer scores at pre-treatment served as a covariate in each model. ADCH = Adherence Checklist, CBCL = Child Behavior Checklist, DASS = Depression Anxiety Stress Scales, DCL-DBD = Diagnostic Checklist for Disruptive Behavior Disorders, FAVK = Questionnaire for Aggressive Behavior of Children, ICU = Inventory of Callous-Unemotional Traits, SCL-ADHD = Symptom Checklist for Attention-Deficit/Hyperactivity Disorder, SCL-DBD = Symptom Checklist for Disruptive Behavior Disorders, ScouT = Computer-Assisted Social Skills Training for Children with Aggressive Behavior, STARK = Resource Activation Treatment, WFIRS-P-M = Weiss Functional Impairment Rating Scale–Parent Report, modified version. β = standardized regression coefficient, *p* = significance level.

^1^ 0 = no, 1 = yes.

^2^ 0 = no qualifications, 1 = lower-track secondary school, 2 = medium-track secondary school, 3 = higher-track secondary school, 4 = university degree.

^3^ 0 = living with one or no primary caregiver (e.g., single parent, residential placement), 1 = living with two primary caregivers.

^a^ STARK *n* = 48, ScouT *n* = 48. ^b^ STARK *n* = 45, ScouT *n* = 48. ^c^ STARK *n* = 50, ScouT *n* = 48. ^d^ STARK *n* = 47, ScouT *n* = 49. ^e^ STARK *n* = 49, ScouT *n* = 48.

Table S3 Prediction of Post-Treatment Child-Rated Peer-Related Aggression (FAVK Total Peer) Separately for STARK and ScouT (*N* = 67)

|  | ScouT | | STARK | |
| --- | --- | --- | --- | --- |
| Predictors | *β* | *p* | *β* | *p* |
| Child characteristics |  |  |  |  |
| Age | -0.13 | .448 | -0.28 | .096 |
| IQ | 0.14 | .396 | 0.05 | .769 |
| Medication^1, a^ | -0.13 | .470 | -0.17 | .354 |
| Sociodemographic characteristics |  |  |  |  |
| Highest parental educational attainment^2, b^ | 0.28 | .093 | 0.36 | .045 |
| Living arrangement^3^ | -0.11 | .507 | -0.08 | .637 |
| Number of children in current household | 0.08 | .625 | -0.22 | .199 |
| Symptom severity |  |  |  |  |
| Parent-rated SCL-DBD Total | -0.00 | .985 | -0.17 | .353 |
| Parent-rated ICU Total | -0.06 | .714 | 0.25 | .144 |
| Parent-rated WFIRS Total | 0.04 | .836 | 0.14 | .403 |
| Clinician-rated DCL-DBD Total | 0.03 | .879 | -0.22 | .197 |
| Comorbid symptoms |  |  |  |  |
| Parent-rated CBCL Internalizing | 0.18 | .292 | -0.09 | .634 |
| Parent-rated SCL-ADHD Total | 0.13 | .439 | 0.28 | .101 |
| Parental symptoms |  |  |  |  |
| Parent-rated DASS Total | 0.20 | .217 | 0.09 | .601 |
| Child Treatment Adherence |  |  |  |  |
| Clinician-rated ADCH^c^ | -0.05 | .767 | 0.12 | .499 |

*Note:* Most potential predictors and moderators were assessed at pre-treatment; only adherence reflects the mean across all 16 sessions. All analyses were conducted separately for each predictor and FAVK Total Peer scores at pre-treatment served as a covariate in each model. ADCH = Adherence Checklist, CBCL = Child Behavior Checklist, DASS = Depression Anxiety Stress Scales, DCL-DBD = Diagnostic Checklist for Disruptive Behavior Disorders, FAVK = Questionnaire for Aggressive Behavior of Children, ICU = Inventory of Callous-Unemotional Traits, SCL-ADHD = Symptom Checklist for Attention-Deficit/Hyperactivity Disorder, SCL-DBD = Symptom Checklist for Disruptive Behavior Disorders, ScouT = Computer-Assisted Social Skills Training for Children with Aggressive Behavior, STARK = Resource Activation Treatment, WFIRS-P-M = Weiss Functional Impairment Rating Scale–Parent Report, modified version. β = standardized regression coefficient, *p* = significance level.

^1^ 0 = no, 1 = yes.

^2^ 0 = no qualifications, 1 = lower-track secondary school, 2 = medium-track secondary school, 3 = higher-track secondary school, 4 = university degree.

^3^ 0 = living with one or no primary caregiver (e.g., single parent, residential placement), 1 = living with two primary caregivers.

^a^ STARK *n* = 33, ScouT *n* = 31. ^b^ STARK *n* = 31, ScouT *n* = 32. ^c^ STARK *n* = 33, ScouT *n* = 31.

Table S4 Hierarchical Multiple Regression to Predict Post-Treatment Teacher-Rated Peer-Related Aggression (FAVK Total Peer; *N* = 98)

|  |  |  |  | 95% CI | |  |  |
| --- | --- | --- | --- | --- | --- | --- | --- |
| Model | | *b* | *SE* | *LL* | *UL* | β | *p* |
| 1 | Intercept | 1.43 | 0.09 | 1.26 | 1.61 |  | <.001 |
|  | Group^1^ | -0.08 | 0.13 | -0.33 | 0.17 | -0.06 | .545 |
|  | adj. *R²* = -.007, adj. Δ*R²* = -.007, Δ*F* (1, 96) = 0.37, *p* = .545 | | | | | | |
| 2 | Intercept | 1.04 | 0.22 | 0.61 | 1.48 |  | <.001 |
|  | Treatment group^1^ | -0.10 | 0.12 | -0.35 | 0.15 | -0.08 | .421 |
|  | Parent-rated FAVK Total Peer | 0.24 | 0.12 | -0.00 | 0.49 | 0.20 | .054 |
|  | adj. *R²* = .022, adj. Δ*R²* = .029, Δ*F* (1, 95) = 3.80, *p =* .054 | | | | | | |
| 3 | Intercept | 1.43 | 0.34 | 0.75 | 2.11 |  | <.001 |
|  | Group^1^ | -0.08 | 0.13 | -0.32 | 0.17 | -0.06 | .547 |
|  | Parent-rated FAVK Total Peer | 0.21 | 0.13 | -0.04 | 0.46 | 0.17 | .094 |
|  | Living arrangement^2^ | -0.19 | 0.13 | -0.46 | 0.07 | -0.15 | .151 |
|  | adj*. R²* = .033, adj. *ΔR²* = .009, Δ*F* (1, 94) = 2.10, *p* = .151 | | | | | | |

*Note*: All predictors were assessed at pre-treatment. Pre-treatment teacher-rated FAVK Peer Total served as a covariate in all models. CBCL = Child Behavior Checklist, DASS = Depression Anxiety Stress Scales, DCL-DBD = Diagnostic Checklist for Disruptive Behavior Disorders, FAVK = Questionnaire for Aggressive Behavior of Children, LL = lower limit, Group = STARK (0) vs. ScouT (1), SCL-ADHD = Symptom Checklist for Attention-Deficit/Hyperactivity Disorder, UL = upper limit, WFIRS = Weiss Functional Impairment Rating Scale–Parent Report, modified version. *b* = unstandardized regression coefficient, β = standardized regression coefficient, *p* = significance level, adj. *R²* = adjusted proportion of explained variance, adj. Δ*R²* = adjusted incremental variance, *SE* = standard error.

^1^ 0 = STARK, 1 = ScouT.

^2^ 0 = living with one or no primary caregiver (e.g., single parent, residential placement), 1 = living with two primary caregivers.

Table S5 Hierarchical Multiple Regression to Predict Post-Treatment Child-Rated Peer-Related Aggression (FAVK Total Peer; *N* = 63)

|  |  |  |  | 95% CI | |  |  |
| --- | --- | --- | --- | --- | --- | --- | --- |
| Model | | *b* | *SE* | *LL* | *UL* | β | *p* |
| 1 | Intercept | 0.58 | 0.07 | 0.44 | 0.71 |  | <.001 |
|  | Group^1^ | -0.05 | 0.10 | -0.24 | 0.14 | -0.07 | .574 |
|  | adj. *R²* = -.011, adj. Δ*R²* = -.011, Δ*F* (1, 61) = 0.32, *p* = .574 | | | | | | |
| 2 | Intercept | 0.33 | 0.10 | 0.12 | 0.54 |  | .003 |
|  | Group^1^ | -0.10 | 0.09 | -0.28 | 0.08 | -0.14 | .273 |
|  | Parent-rated FAVK Total Peer | 0.29 | 0.10 | 0.10 | 0.48 | 0.37 | .004 |
|  | adj. *R²* = .108, adj. Δ*R²* = .119, Δ*F* (1, 60) = 9.15, *p =* .004 | | | | | | |
| 3 | Intercept | 0.07 | 0.13 | -0.20 | 0.34 |  | .621 |
|  | Group^1^ | -0.12 | 0.09 | -0.29 | 0.05 | -0.16 | .172 |
|  | Parent-rated FAVK Total Peer | 0.28 | 0.09 | 0.10 | 0.46 | 0.36 | .003 |
|  | Highest parental educational attainment^2^ | 0.11 | 0.04 | 0.03 | 0.19 | 0.32 | .006 |
|  | adj. *R²* = .202, adj. Δ*R²* = .094, Δ*F* (1, 59) = 8.08, *p* = .006 | | | | | | |

*Note*: All predictors were assessed at pre-treatment. Pre-treatment child-rated FAVK Peer Total served as a covariate in all models. CBCL = Child Behavior Checklist, DASS = Depression Anxiety Stress Scales, DCL-DBD = Diagnostic Checklist for Disruptive Behavior Disorders, FAVK = Questionnaire for Aggressive Behavior of Children, LL = lower limit, Group = STARK (0) vs. ScouT (1), SCL-ADHD = Symptom Checklist for Attention-Deficit/Hyperactivity Disorder, UL = upper limit, WFIRS = Weiss Functional Impairment Rating Scale–Parent Report, modified version. *b* = unstandardized regression coefficient, β = standardized regression coefficient, *p* = significance level, adj. *R²* = adjusted proportion of explained variance, adj. Δ*R²* = adjusted incremental variance, *SE* = standard error.

^1^ 0 = STARK, 1 = ScouT.

^2^ 0 = no qualifications, 1 = lower-track secondary school, 2 = medium-track secondary school, 3 = higher-track secondary school, 4 = university degree.

Table S6 Moderation of the Effects of ScouT Compared to STARK on Post-Treatment Parent-Rated Peer-Related Aggression (FAVK Total Peer; *N* = 100)

|  | *b* | | *SE* | *t* | *p* | 95% CI | | |
| --- | --- | --- | --- | --- | --- | --- | --- | --- |
|  |  |  |  |  |  | *LL* | *UL* | |
| Group | -0.96^a^ | 0.51 | | -1.87 | .064 | -1.97 | | 0.06 |
| Age | -0.08 | 0.04 | | -2.39 | .019 | -0.15 | | -0.01 |
| Group x Age | 0.06 | 0.05 | | 1.24 | .218 | -0.04 | | 0.16 |
| Parent-rated FAVK Total Peer Pre | 0.59 | 0.11 | | 5.30 | < .001 | 0.37 | | 0.81 |
| Group | -1.52^a^ | 0.71 | | -2.14 | .035 | -2.92 | | -0.11 |
| IQ | -0.01 | 0.00 | | -2.09 | .039 | -0.02 | | -0.00 |
| Group x IQ | 0.01 | 0.01 | | 1.67 | .098 | -0.00 | | 0.03 |
| Parent-rated FAVK Total Peer Pre | 0.60 | 0.11 | | 5.34 | < .001 | 0.03 | | 0.83 |
| Group | -0.25 | 0.38 | | -0.64 | .523 | -1.01 | | 0.52 |
| Parent-rated SCL-DBD Total | 0.46 | 0.41 | | 1.14 | .259 | -0.34 | | 1.27 |
| Group x Parent-rated SCL-DBD Total | -0.08 | 0.45 | | -0.17 | .864 | -0.98 | | 0.82 |
| Parent-rated FAVK Total Peer Pre | 0.46 | 0.15 | | 3.16 | .002 | 0.17 | | 0.75 |
| Group | -1.08 | 0.38 | | -2.88 | .005 | -1.83 | | -0.34 |
| Clinician-rated DCL-DBD Total | -0.10 | 0.28 | | -0.36 | .723 | -0.66 | | 0.46 |
| Group x Clinician-rated DCL-DBD Total | 0.85 | 0.39 | | 2.19 | .031 | 0.08 | | 1.61 |
| Parent-rated FAVK Total Peer Pre | 0.52 | 0.12 | | 4.49 | <.001 | 0.29 | | 0.75 |
| Group | -0.64 | 0.23 | | -2.74 | .007 | -1.10 | | -0.18 |
| Parent-rated WFIRS Total | 0.30 | 0.16 | | 1.90 | .060 | -0.01 | | 0.62 |
| Group x WFIRS Total | 0.37 | 0.22 | | 1.71 | .091 | -0.06 | | 0.81 |
| Parent-rated FAVK Total Peer Pre | 0.41 | 0.11 | | 3.62 | .001 | 0.18 | | 0.63 |
| Group | -0.54 | 0.16 | | -3.41 | .001 | -0.85 | | -0.23 |
| Parent-rated CBCL Internalizing | 0.53 | 0.28 | | 1.91 | .059 | -0.02 | | 1.08 |
| Group x Parent-rated CBCL Internalizing | 0.83 | 0.40 | | 2.10 | .039 | 0.04 | | 1.61 |
| Parent-rated FAVK Total Peer Pre | 0.41 | 0.11 | | 3.71 | <.001 | 0.19 | | 0.63 |
| Group | -0.30 | 0.21 | | -1.40 | .165 | -0.72 | | 0.13 |
| Parent-rated SCL-ADHD Total | 0.27 | 0.10 | | 2.73 | .007 | 0.07 | | 0.47 |
| Group x Parent-rated SCL-ADHD Total | 0.03 | 0.14 | | 0.20 | .842 | -0.26 | | 0.31 |
| Parent-rated FAVK Total Peer Pre | 0.49 | 0.11 | | 4.46 | <.000 | 0.27 | | 0.71 |
| Group | -0.66 | 0.42 | | -1.55 | .126 | -1.50 | | 0.19 |
| Parent-rated DASS Total | 0.24 | 0.22 | | 1.07 | .288 | -0.20 | | 0.67 |
| Group x Parent-rated DASS Total | 0.22 | 0.28 | | 0.79 | .432 | -0.34 | | 0.79 |
| Parent-rated FAVK Total Peer Pre | 0.55 | 0.11 | | 4.95 | <.000 | 0.33 | | 0.77 |

*Note:* Pre-treatment parent-rated FAVK Peer Total served as a covariate in all analyses. CBCL = Child Behavior Checklist, DASS = Depression Anxiety Stress Scales, DCL-DBD = Diagnostic Checklist for Disruptive Behavior Disorders, FAVK = Questionnaire for Aggressive Behavior of Children, Group = STARK (0) vs. ScouT (1), LL = lower limit, Pre = pre-treatment, SCL-ADHD = Symptom Checklist for Attention-Deficit/Hyperactivity Disorder, SCL-DBD = Symptom Checklist for Disruptive Behavior Disorders, ScouT = Computer-Assisted Social Skills Training for Children with Aggressive Behavior, STARK = Resource Activation Treatment, UL = upper limit, WFIRS-P-M = Weiss Functional Impairment Rating Scale–Parent Report, modified version, *b* = unstandardized coefficient for the interaction effect, *CI* = confidence interval, *n* = sample size, *p* = significance value, *SE* = standard error, *t* = empirical *t*-value.

^a^ As the moderator lacks a meaningful zero point and is not mean-centered, caution is warranted when interpreting this coefficient.

Table S7 Moderation of the Effects of ScouT Compared to STARK on Post-Treatment Teacher-Rated Peer-Related Aggression (FAVK Total Peer; *N* = 98)

|  | *b* | | *SE* | | *t* | | *p* | | 95% CI | | | *f^2^* | |
| --- | --- | --- | --- | --- | --- | --- | --- | --- | --- | --- | --- | --- | --- |
|  |  |  |  |  |  |  |  |  | *LL* | | *UL* |  |  |
| Group^1^ | 0.59 | 0.30 | | 1.94 | | .055 | | -0.01 | | 1.19 | | |  |
| Living arrangement^2^ | 0.04 | 0.20 | | 0.19 | | .851 | | -0.37 | | 0.44 | | |  |
| Group x Living arrangement^2, a^ | -0.78 | 0.33 | | -2.39 | | .019 | | -1.44 | | -0.13 | | | 0.06 |
| Parent-rated FAVK Total Peer Pre | 0.19 | 0.12 | | 1.59 | | .115 | | -0.05 | | 0.44 | | |  |

*Note:* Pre-treatment teacher-rated FAVK Peer Total served as a covariate in all analyses. FAVK = Questionnaire for Aggressive Behavior of Children, Group = STARK (0) vs. ScouT (1), LL = lower limit, Pre = pre-treatment, ScouT = Computer-Assisted Social Skills Training for Children with Aggressive Behavior, STARK = Resource Activation Treatment, UL = upper limit, *b* = unstandardized coefficient for the interaction effect, *CI* = confidence interval, *p* = significance value, *SE* = standard error, *t* = empirical *t*-value.

^1^ 0 = STARK, 1 = ScouT.

^2^ 0 = living with one or no primary caregiver (e.g., single parent, residential placement), 1 = living with two primary caregivers.

Figure S1 Living Arrangement as a Moderator of the Effects of ScouT Compared to STARK on Post-Treatment Teacher-Rated Peer-Related Aggression (FAVK Total Peer; *N* = 98)

*Note*. The pre-treatment teacher-rated FAVK Total Peer served as a covariate. FAVK = Questionnaire for Aggressive Behavior of Children, ScouT = Computer-Assisted Social Skills Training for Children with Aggressive Behavior, STARK = Resource Activation Treatment.

Table S8 Moderation of the Effects of ScouT Compared to STARK on Post-Treatment Child-Rated Peer-Related Aggression (FAVK Total Peer; *N* = 63)

|  | *b* | | *SE* | | *t* | | *p* | | 95% CI | | | *f^2^* | |
| --- | --- | --- | --- | --- | --- | --- | --- | --- | --- | --- | --- | --- | --- |
|  |  |  |  |  |  |  |  |  | *LL* | | *UL* |  |  |
| Group^1^ | -0.11 | 0.21 | | -0.53 | | .601 | | -0.54 | | 0.32 | | |  |
| Highest parental educational attainment^2^ | 0.11 | 0.05 | | 2.08 | | .042 | | 0.00 | | 0.22 | | |  |
| Group x Highest parental educational attainment^2^ | -0.00 | 0.08 | | -0.03 | | .975 | | -0.16 | | 0.16 | | | 0.00 |
| Parent-rated FAVK Total Peer Pre | 0.28 | 0.09 | | 3.10 | | .003 | | 0.10 | | 0.47 | | |  |

*Note:* Pre-treatment child-rated FAVK Peer Total served as a covariate in all analyses. FAVK = Questionnaire for Aggressive Behavior of Children, Group = STARK (0) vs. ScouT (1), LL = lower limit, Pre = pre-treatment, ScouT = Computer-Assisted Social Skills Training for Children with Aggressive Behavior, STARK = Resource Activation Treatment, UL = upper limit, *b* = unstandardized coefficient for the interaction effect, *CI* = confidence interval, *p* = significance value, *SE* = standard error, *t* = empirical *t*-value.

^1^ 0 = STARK, 1 = ScouT.

^2^ 0 = no qualifications, 1 = lower-track secondary school, 2 = medium-track secondary school, 3 = higher-track secondary school, 4 = university degree.
